# FOOD INTAKE EVALUATION DURING THE FIRST YEAR OF POSTOPERATIVE OF PATIENTS WITH TYPE 2 DIABETES MELLITUS OR GLYCEMIC ALTERATION SUBMITTED TO ROUX-EN-Y GASTRIC BYPASS

**DOI:** 10.1590/0102-672020180001e1367

**Published:** 2018-07-02

**Authors:** Marilia R. ZAPAROLLI, Magda R. R. DA-CRUZ, Caroline FREHNER, Alcides J. BRANCO-FILHO, Maria Eliana M. SCHIEFERDECKER, Antônio C. L. CAMPOS, Cesar Augusto TACONELI, Guilherme PARREIRA

**Affiliations:** 1 Departamento de Nutrição, Universidade Federal do Paraná; 2 Programa de Pós- Graduação em Alimentação e Nutrição, Universidade Federal do Paraná; 3 Departamento de Cirurgia, Universidade Federal do Paraná; 4 Departamento de Estatística, Universidade Federal do Paraná; 5 Cevip-Paraná, Centro de Laparoscopia, Curitiba, PR, Brasil

**Keywords:** Gastric bypass, Nutrients, Food consumption., Derivação gástrica, Nutrientes, Ingestão de alimentos

## Abstract

***Background*:**

*:* Obesity is one of the main causes of glycemic change. Failure of clinical obesity treatment may lead to an increase in bariatric surgery. Dietary guidance, in conjunction with disabsorptive and hormonal factors resulting from the anatomical and physiological changes provoked by the surgery, is associated with changes in food intake.

***Aim*:**

To analyze food intake evolution during the first postoperative year of Roux-en-y gastric bypass in patients with type 2 diabetes mellitus or glycemic alteration.

***Methods*:**

*:* This was a longitudinal and retrospective observational study. For food intake evolution analysis, linear regression models with normal errors were adjusted for each of the nutrients.

***Results*:**

At 12 months, all patients presented improvement in glycemic levels (p&lt;0.05). During the first postoperative year, there was a reduction in energy intake, macronutrients, consumption of alcoholic beverages and soft drinks. Conversely, there was an increase in fiber intake and diet fractionation. It was observed that, despite gastric restrictions, the micronutrient intake specifically recommended for glycemic control was greater up to six months postoperatively.

***Conclusion*:**

There was change in the quantity and quality of food intake. It was the most prevalent glycemic control contributor up to six months postoperatively. At the end of one year, the diet underwent a change, showing a similar tendency to the preoperative food intake pattern.

## RESUMO

***Racional*:**

Obesidade é uma das maiores causas de alteração glicêmica. O insucesso no seu tratamento clínico pode levar ao aumento de operações bariátricas. Orientação dietética, em conjunto com fatores disabsortivos e hormonais resultantes das alterações anatômicas e fisiológicas provocadas pela operação, está associada à mudanças na ingestão alimentar.

***Objetivo*:**

*:* Analisar a evolução da ingestão alimentar durante o primeiro ano pós-operatório de bypass gástrico em Y-de-Roux de pacientes com diabete *melito* tipo 2 ou alteração glicêmica no pré-operatório.

*:* Estudo observacional longitudinal e retrospectivo. Para análise da evolução da ingestão alimentar, modelos de regressão linear com erros normais foram ajustados para cada nutriente.

***Resultados*:**

Aos 12 meses, todos os pacientes apresentaram melhora nos níveis de glicemia (p&lt;0,05). Durante o primeiro ano pós-operatório, houve redução na ingestão de energia, macronutrientes, consumo de bebidas alcoólicas e refrigerantes. Por outro lado, houve aumento na ingestão de fibras e fracionamento de dieta. Observou-se que, apesar das restrições gástricas, a ingestão de micronutrientes recomendados especificamente para o controle glicêmico foi maior até seis meses do pós-operatório.

***Conclusão*:**

Houve mudança na quantidade e na qualidade da ingestão alimentar, sendo o consumo de alimentos que contribuem para o controle glicêmico mais prevalente até o sexto mês de pós-operatório. No pós-operatório em até um ano, a dieta sofre alteração, apresentando inadequações em relação à pirâmide específica, com tendência ao padrão alimentar do pré-operatório.

## INTRODUCTION

Obesity is the major cause of glycemic changes and progression to type 2 diabetes mellitus (DM2)^17^. It is estimated that 44% of cases are correlated with overweight^27^. 

The dietary intake profile is directly associated with glycemic improvement. The goal is to maintain glycemic levels within or near normality^21^. Therefore, energy and fat reduction, as well as the reduction or exclusion of sugary drink intake and increased fiber consumption, are essential strategies for glycemic improvement^1^ ^,^ ^3^ ^,^ ^4^.

Daily intake of fruits and vegetables provides a high amount of micronutrients with potential antioxidant effect, minimizing the damage caused by oxidative and metabolic stress related to glycemic changes^5^. Zinc, magnesium, selenium, and antioxidant vitamins, especially vitamin C, can directly affect glucose homeostasis^26^.

In the postoperative period of bariatric surgery, we previously observed a change in the pattern of food intake through a significant reduction in energy intake and that of micro and macronutrients^18^. Additionally, bariatric surgery has been associated with behavioral changes related to food preference and choice, as well as increasing dietary fractionation^12^. 

Therefore, this study aimed to analyze food intake evolution during the first postoperative year of Roux-en-y gastric bypass (RYGB) in patients with DM2 or glycemic alteration.

## METHODS

This was a retrospective, longitudinal, observational, analytical study. It was carried out after approval by the Research Ethics Committee of the Pontifical Catholic University of Paraná. The study was registered under identifier 13491913.8.0000.0020.

A convenience sampling method was used, and only subjects who met the inclusion criteria were selected. The inclusion criteria were as follows: adult and elderly patients of both genders, diagnosed with DM2 or fasting hyperglycemia confirmed by preoperative biochemical tests (fasting glycemia and HbA1c), who underwent RYGB between 2007 and 2014. Medical data included nutritional care records at three, six and 12 months preoperatively.

The following criteria were considered as diagnostic criteria for DM2 and fasting hyperglycemia: DM2 was diagnosed if fasting glucose ≥126 mg/dl or HbA1c ≥6.5%; fasting hyperglycemia was diagnosed if fasting glucose ranged between 100-125 mg/dl or HbA1c between 5.7% and 6.4%^2^ ^,^ ^24^. 

All the information was collected from electronic medical record and was recorded by the investigator (nutritionist). Physical activity was defined as performing at least 150 min of moderate intensity physical exercise per week^27^. Food intake data were evaluated based on the 24-hour recall (R24h) estimation in each established periods and for soft drink and alcoholic beverage it was used weekly consumption frequency. For diet analysis, ADS Nutri® software was used.

### Statistical analysis

Descriptive statistics were used to describe the data. To compare the pre- and postoperative periods in terms of DM2 evolution, was used the MacNemar statistical test. For food intake evolution analysis, linear regression models with normal errors were adjusted for each of the nutrients considered^6^. The R NLME software package was used considering a significance level of 5%.

## RESULTS

A total of 754 nutritional monitoring records were reviewed among the medical records of patients operated between 2007 and 2014, and the records of patients that met the inclusion criteria were selected. The final sample consisted of 106 patients. However, in the 3-month and 6-month interim consultations, data were only available for 100 and 98 patients, respectively. Considering nutritional consultations as an indicator of treatment adherence, the treatment adherence was 94.3% at three months, 92.5% at six months, and 100% at 12 months postoperatively.

In the study sample, 90.5% (n=96) of patients were females. Patients had a mean (range) age of 48 (20-64) years. Regarding preoperative obesity, 67.9% (n=72) presented this clinical condition for 10 years or more. When investigating the family history of obesity and DM2, 87.7% (n=93) reported relatives with obesity and 75.5% (n=80) had relatives with DM2. The majority of patients (61.3%, n=65), reported a diagnosis of three or more comorbidities in the preoperative period (Table 1).

**TABLE 1:** Sample characterization

Variable	Pre-operative	3 months PO	6 months PO	12 months PO
Sample (n)	106	100	98	106
Gender				
Female % (n)	90.5 (96)	90 (90)	92.8 (91)	90.5 (96)
Male % (n)	9.5 (10)	10 (10)	7.2 (7)	9.5 (10)
Age (years)*	48 (20-64)	48 (20-64)	47 (20-64)	48 (20-64)
Obesity history (years)				
≤10 years	32.1 (34)	NA	NA	NA
&gt;10 years	67.9 (72)	NA	NA	NA
Family medical history of obesity				
Yes % (n)	87.7 (93)	NA	NA	NA
No % (n)	12.3 (13)	NA	NA	NA
Family medical history of DM2				
Yes % (n)	75.5 (80)	NA	NA	NA
No % (n)	24.5 (26)	NA	NA	NA
Comorbidities				
Two comorbidities % (n)	38.7 (41)	23 (23)	13.2 (13)	6.6 (7)
Three or more comorbidities % (n)	61.3 (65)	NA	NA	NA

*=values are expressed in median (minimum value-maximum value) descriptive statistics (software R); PO=postoperative; DM2=type 2 diabetes mellitus; NA=not applicable; %=percentage in relation to the sample; n = number of patients

The preoperative BMI was 39.6 (32.8-67.8). At three months, patients had a mean BMI of 31.5 (23.9-53.3) kg/m^2^. At six and 12 months, the mean BMI was 28.1 (22.3-49.9) kg/m^2^ and 26.8 (19.0-48.5) kg/m^2^, respectively. The percentage of excess weight loss increased from 53.7% (23.8-112.5) at three months postoperatively to 72.8% (33.2-139.4) at six months, and 87.8% (36.2-150.4) at 12 months. The absolute weight loss was 21.4 (8.8-44.5) kg, 28.5 (13.8-73) kg and 32.4 (21.5-88) kg at three, six and 12 months postoperatively, respectively.

In the preoperative period, 51.8% (n=55) of the patients presented DM2 and 48.2% (n=51) had fasting hyperglycemia. Despite the glycemic alterations, only 32.1% (n=34) used hypoglycemic drugs and/or insulin.

### Food Intake

It was estimated that energy, that is, caloric intake, at three months of surgery, represented, on average, 35.7% of the preoperative caloric intake (p&lt;0.05). At six months and 12 months postoperatively, this estimate increased to 40.3% and 49.7% of the preoperative caloric intake, respectively (p&lt;0.05). It was observed that the ingested caloric value presented similar results for all age categories, years of obesity, classification of BMI, number of comorbidities and use of drugs.

Regarding carbohydrate intake, it was estimated that, at three months postoperatively, patients consumed, on average, 35.3% of carbohydrates ingested in the preoperative period (p&lt;0.05). At six and 12 postoperatively, this estimate increased to 39.4% and 49.4%, respectively (p&lt;0.05). Male patients presented a carbohydrate consumption 12.3% higher than female patients (p&lt;0.05).

At three months postoperatively, lipid intake represented, on average, 26.3% of the preoperative lipid intake (p&lt;0.05). At six months, the mean was 31.8% of the initial consumption and at 12 months, this value increased to 39.5% (p&lt;0.05). The lipid values were similar in all age categories, duration of obesity, classification of BMI, number of comorbidities and use of medications.

At three months postoperatively, magnesium consumption represented 61.3% of preoperative consumption (p&lt;0.05). At six and 12 months, consumption corresponded to 66.7% and 79.3%, respectively (p&lt;0.05). It was observed that patients with a history of obesity greater than 10 years, presented an average consumption of 9.19% less magnesium than the others (p&lt;0.05).

The zinc intake at three months postoperatively corresponded to 6.73 mg/day less compared with the preoperative period. At six and 12 months, a lower consumption was observed at 5.39 mg/day and 4.83 mg/day, respectively, compared with the preoperative period (p&lt;0.05). The amounts ingested at dix and 12 months were similar to each other (p&gt;0.05).

In the preoperative period, the majority of patients (87.7%) reported a consumption of three to five meals per day, and only 10.4% (n=11) patients fractionated the diet in six or more meals. A significant change was observed in the fractionation: 76% (n=76), 78.6% (n=77) and 72.7% (n=77) of patients reported fractionation of 6 or more meals/daily.

In the preoperative period, there was a high prevalence of soft drink consumption (65.1%). For alcoholic beverages, the prevalence was lower, representing 13.2% (n=14) of the sample. At three months postoperatively, no patient reported consumption of soft drinks or alcoholic beverages. However, at six postoperatively, 6.1% drank soda and alcohol. At 12 months, the intake of soft drinks increased to 14.6% while the consumption of alcoholic beverages increased to 11.3%.

**TABLE 2:** Food intake analysis

Variables	3 months PO	6 months PO	12 months PO
Energy (kcal/day)			
Estimate	-1.028	-0.909	-0.698
Default error	0.026	0.029	0.03
Exponential	0.358 ^a^	0.403 ^b^	0.497 ^c^
Carbohydrate (g/day)			
Estimate	-1.042	-0.931	-0.705
Default error	0.032	0.036	0.037
Exponential	0.353 ^a^	0.394 ^b^	0.494 ^c^
Protein (g/day)			
Estimate	-39.930 ^a^	-35.710 ^b^	-28.280 ^c^
Default error	3.084	3.291	3.337
Lipids (g/day)			
Estimate	-1.336	-1.146	-0.929
Default error	0.055	0.005	0.051
Exponential	0.263 ^a^	0.318 ^b^	0.395 ^c^
Fiber (g/day)			
Estimate	0.677 ^a^	1.596 ^b^	4.939 ^c^
Default error	0.378	0.396	0.539
Zinc (mg/day)			
Estimate	-6.735 ^a*^	-5.390 ^b^	-4.832 ^b^
Default error	0.824	0.824	0.824
Magnesium (mg/day)			
Estimate	-0.49	-0,405	-0.232
Default error	0.042	0.047	0.048
Exponential	0.613 ^a^	0.667 ^b^	0.793 ^c^

Statistical test used=mixed regression model; exponential corresponds to the exponential value of the estimate; different letters mean p&lt;0.05; PO=postoperative; all postoperative data were compared with preoperative without adjustment; *when compared with the preoperative, value p&gt; 0.05

## DISCUSSION

In parallel with the current global obesity epidemic, the incidence of DM2 is also increasing. It is known that approximately 23% of patients with morbid obesity present with DM2, and only 8% are diagnosed at the initial stage of this clinical condition^10^ ^,^ ^29^. Conventional medical treatment for glycemic control is challenging because some oral hypoglycemic agents and insulin may result in weight gain. As an alternative treatment, bariatric surgery has become an effective treatment to achieve remission of DM2 and other related comorbidities, reducing cardiovascular risk and consequently the number of obesity-related deaths^14^. 

The multidisciplinary treatment adherence is one of the factors that influences the clinical and nutritional evolution of patients. The high adherence levels can be justified by the fact that the data collection was performed at the Bariatric Surgery Excellence Clinic, which has a health monitoring rate of at least 75% of the individuals for a period of five years of postoperative follow-up^25^. However, the rate of multidisciplinary adherence follow-up tends to decrease over the years. This fact raises several concerns given that surgical results are associated with dietary and behavioral factors that extend to the late postoperative period. Recommendations regarding food intake and eating behaviors are predictive of clinical evolution from the immediate to the late period. Sarwer et al. showed that adherence guidelines applied at 20 weeks postoperatively translated into weight loss at 96 weeks after surgery^22^.

Regarding the food intake analysis, this study showed that added to the quantitative change, there was a change in diet quality. When compared with the preoperative period, the postoperative diet was characterized by a reduced consumption of energy intake, carbohydrates, proteins, lipids, alcoholic beverages, and soft drinks. Conversely, there was an increase in fiber intake and diet fractionation. Regarding micronutrients specific for glycemic control, such as zinc and magnesium, it was observed that despite the gastric restriction, food sources of these nutrients were present more frequently postoperatively.

Recent studies have presented new insights on postoperative glycemic improvement, including decrease of energy consumption as a major recommendation for rapid improvement in blood glucose levels. In addition to the mechanisms responsible for appetite suppression, there was a change in the food choice; the most observed substitution was the consumption of sweet foods with high energy density by foods of low energy density16. In the study of taste perception and taste alteration after RYGB, some authors concluded that there is an increase in the stimuli to bitter and acidic flavors and a reduction in the sweet flavor stimuli^20^ ^,^ ^28^ ^,^ ^30^.

Additionally, the change from preoperative to postoperative food patterns was associated with diet guidance. Changing dietary habits can decrease the risk of postoperative complications, ensuring surgical success^7^. In the late postoperative period, the adapted food pyramid facilitates the choice of food, allowing for a balanced diet, with adequate variety and proportionality of food groups^15^. Another factor associated with postoperative food choices is the so-called dumping syndrome, in which patients stop consuming high-calorie or high-fat foods and sugars, and increase their intake of high-fiber foods for fear of presenting complications^8^ ^,^ ^9^.

Despite the improvement in the postoperative food pattern in relation to the preoperative period, when analyzing the evolution of the diet during the three, six and 12 postoperative months, it was observed that the adequate consumption of foods that contribute to glycemic control is more prevalent up to six months postoperatively. This fact suggests a higher concern of patients in the immediate postoperative period, when the diet has specific characteristics, reflecting the lack of understanding of the importance of food re-education in long-term surgical success^13^.

For analysis of food intake, R24h were used, and these data were adjusted from regression models. Recently, a study with diabetic patients who underwent RYGB showed that R24h is an appropriate instrument to analyze nutrient intake before and after bariatric surgery; however, it is insufficient to evaluate possible long-term deficits^2^. It is known that R24h has several limitations, that is, it depends on the interviewee’s report, and overestimation or underestimation of food consumption may occur^11^. The underestimation is perceived in the preoperative period, because it is a moment where food intake is high and the patient opts for omission. However, there are alternatives to improve the accuracy of food consumption data collected from R24h. Regardless of whether a single datum or more is analyzed, this analysis is susceptible to errors, which can be minimized by the use of an appropriate statistical approach19. In order to minimize these errors, we used several techniques in this study to improve food intake analysis: the data were collected by a single professional in bariatric surgery, who performed a review of the recall for detection of errors or omissions. All the data were coded and their measurements were standardized. We used structured analysis software for scientific research, composed of two tables of reference in the area of food composition.

## CONCLUSION

During the first postoperative year of RYGB, patients with postoperative glycemic improvement presented a change in the quantity and quality of food intake. Food intake was the most prevalent glycemic control contributor up to six months postoperatively. In the late postoperative period, the diet underwent a change, with similar tendencies in food patterns as observed in the preoperative period.
